# Fluorescein Angiography for Monitoring Neural Blood Flow in Chronic Nerve Compression Neuropathy: Experimental Animal Models and Preliminary Clinical Observations

**DOI:** 10.3390/neurolint16050074

**Published:** 2024-09-05

**Authors:** Kosuke Saito, Mitsuhiro Okada, Takuya Yokoi, Shunpei Hama, Hiroaki Nakamura

**Affiliations:** 1 Department of Orthopedic Surgery, Graduate School of Medicine, Osaka City University, 1-4-3, Asahimachi, Abenoku, Osaka 545-8585, Japan; saitokosuke97@gmail.com (K.S.); hnakamura.ocu@gmail.com (H.N.); 2 Department of Orthopedic Surgery, Graduate School of Medicine, Osaka Metropolitan University, 1-4-3, Asahimachi, Abenoku, Osaka 545-8585, Japan; 3 Department of Orthopaedic Surgery, Seikeikai Hospital, 1-1-1, Minami-Yasui-cho, Sakaiku, Sakai, Osaka 590-0064, Japan; takuyayokoi@gmail.com; 4 Department of Orthopaedic Surgery, Yodogawa Christian Hospital, 1-7-50, Kunijima, Higashi Yodogawaku, Osaka 533-0024, Japan; shunpeihama58@gmail.com

**Keywords:** nerve blood flow, chronic nerve compression, fluorescein angiography, laser doppler flowmetry, neuropathy

## Abstract

Pathologies associated with neural blood disturbance have been reported in patients with chronic nerve compression (CNC) neuropathy. Fluorescein angiography (FAG) and laser Doppler flowmetry (LDF) are effective for real-time peripheral nerve blood flow assessment. However, their reliability in severe neuropathy models in large animals or clinical conditions remains unclear. Initially, we aim to apply FAG to two different CNC animal models and evaluate their characteristics in comparison with those of LDF. In FAG, we quantified the peak luminance at the compression site following fluorescein injection. Then, we positioned the LDF probe at the center of the compression site and recorded the blood flow. Subsequently, we analyzed whether the FAG characteristics obtained in this animal experiment were consistent with those of clinical studies in patients with severe carpal tunnel syndrome (CTS). In the CNC rat model, FAG and LDF effectively monitored reduced neural blood flow over time. We observed significant blood flow reduction using both techniques in a newly developed severe CNC rabbit model. Notably, FAG correlated strongly with the compound muscle action potential (CMAP) amplitude in electrodiagnostic findings, unlike LDF. As a next step, we performed FAG after open carpal tunnel release in clinical cases of CTS. FAG correlated significantly with preoperative CMAP amplitude. This indicates FAG’s importance for assessing nerve blood flow during surgery, potentially improving diagnostic accuracy and surgical outcomes.

## 1. Introduction

Chronic nerve compression (CNC) neuropathies, including carpal tunnel syndrome (CTS) and cubital tunnel syndrome, are common hand conditions [1,2] that cause pain, numbness, and muscle weakness [3]. These conditions may lead to significant economic burdens due to absenteeism and treatment costs [4]. Therefore, the pathogenesis of CNC should be better understood in order to improve diagnosis and treatment outcomes.

The pathology of CNC neuropathies is associated with disturbances in neural blood flow [5,6,7]. Regarding CTS, one of the most common peripheral CNCs [8,9], chronic pressure in the carpal tunnel is reported to compress the median nerve and compromise blood flow [10]. Functional changes in the median nerve can be assessed through electrodiagnostic studies [11,12]. Previous studies have shown that combining NCS with neurovascular blood flow assessment improves diagnostic accuracy [13,14,15,16]. A previous study utilized LDF during carpal tunnel release to observe changes in the microvascular flow in the median nerve [17]. In another study, Hama et al. used FAG in a mouse CNC model to visualize the microvascular blood flow in the affected nerve and reported pathologies associated with nerve fibrosis [18]. They found that blood flow disturbance in the FAG reflected the early pathology of CNC and served as an early marker. This study presents a novel analysis of CNC pathology using FAG. FAG offers advantages such as safety, with few adverse events [19,20], and technical ease in visualizing neural blood flow [18]. We focused on the ability of FAG to visualize areas of reduced blood flow and its potential to improve intraoperative diagnostic accuracy, provided it could ensure diagnostic accuracy equivalent to existing methods of blood flow assessment. However, its potential application in severe CNC models in larger animals or clinical applications compared with LDF remains unclear.

Therefore, our primary aim is to apply FAG to the nerve compression site in two different CNC animal models and compare the results with those of LDF. Subsequently, we analyzed whether the FAG characteristics obtained in this animal experiment were consistent with those of clinical studies in patients with severe CTS.

## 2. Materials and Methods

### 2.1. Animal Models

All animals were maintained under a 24 h light–dark cycle, with ad libitum access to food and water. Before surgery, animals were anesthetized by subcutaneous injection of ketamine (50 mg/mL) (Daiichi Sankyo Healthcare Co. Ltd., Tokyo, Japan) and xylazine (0.2 mg/mL) (Bayer HealthCare, Leverkusen, Germany) in a 10:3 ratio at a dose of 1 mL/kg total body weight. All experimental procedures were approved by the Medicine Committee on Animal Research (approval number: 21101).

#### 2.1.1. Rat CNC Model

Eight-week-old male LEW/SsNSlc inbred rats (Japan SLC Inc., Hamamatsu, Japan) were used for the study. A CNC neuropathy model was established following a previously described protocol [21,22,23].

**Figure 1:**
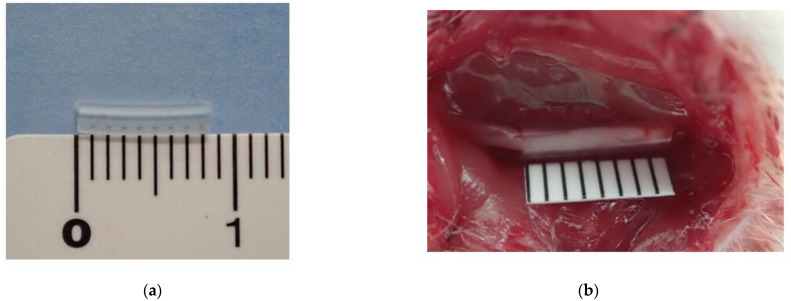
Silastic tube wrapped around rat sciatic nerve. (**a**) An 8 mm silastic tube with an inner diameter of 1.3 mm. (**b**) The silastic tube compresses the sciatic nerve.

The left sciatic nerve was exposed between the quadriceps and biceps femoris muscles using an intermuscular approach. An 8 mm length silastic tube (Baxter Healthcare, Deerfield, MA, USA) with an inner diameter of 1.3 mm was wrapped around the common sciatic nerve (Figure 1). The rats were divided into three groups (n = 6 per group): uninjured (control), 4-week CNC neuropathy, and 8-week CNC neuropathy.

**Figure 2:**
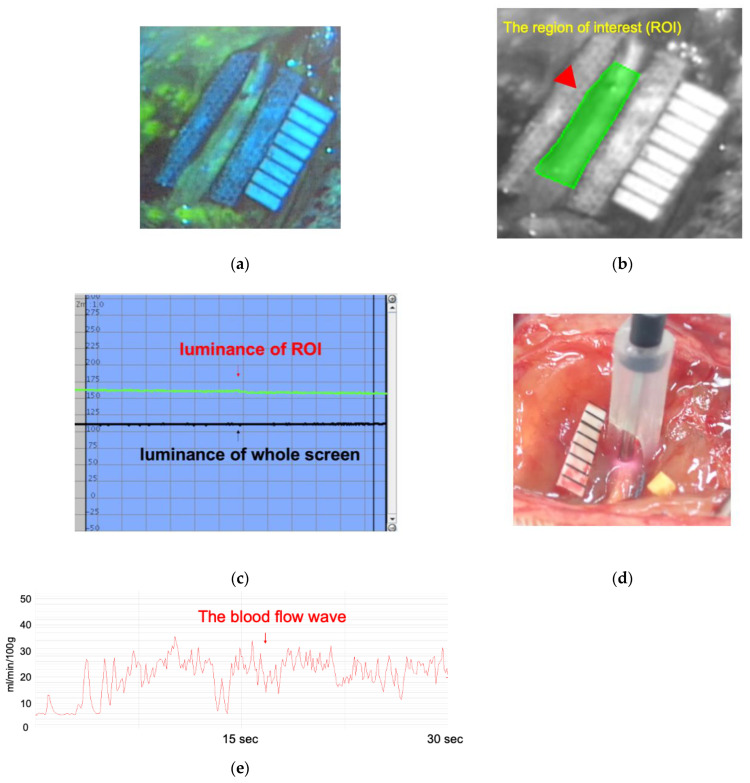
Blood flow assessment by fluorescein angiography (FAG) and laser Doppler flowmetry (LDF) for rat sciatic nerve. (**a**) FAG image recorded under a microscope through a FAG filter. (**b**) Flowinsight software (Version 2.5.0) screen with the region of interest (ROI) on the compressed nerve. The red arrow indicates the ROI. (**c**) The Flowinsight software calculates the fluorescein luminance. (**d**) The LDF probe was positioned on the compression site. (**e**) The blood flow was recorded and calculated by FLO-C1.

We performed LDF and FAG on each group during the removal of the silicone tube from the sciatic nerve during reoperation (Figure 2). This study focused on assessing the correlation between LDF and FAG using a well-established rodent model.

#### 2.1.2. Rabbit Severe Chronic Neuropathy Model

**Figure 3:**
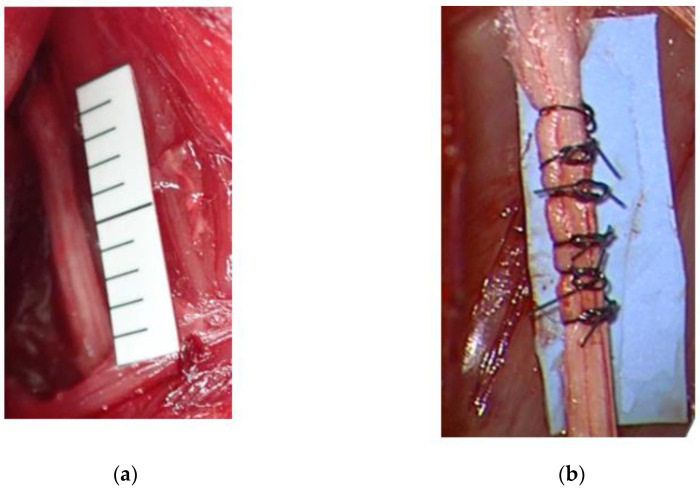
Rabbit severe chronic neuropathy model. (**a**) Common sciatic nerve before compression. (**b**) Sciatic nerve after ligated compression.

Retired female Slc: JW/CSK rabbits (Japan SLC Inc., Hamamatsu, Japan) were used in this experiment to develop a rabbit model of severe chronic neuropathy. Briefly, the left sciatic nerve was exposed, and six ligatures (No. 4-0 nylon sutures) were loosely tied around the nerve at a length of 20 mm (Figure 3). Subsequently, the rabbits were divided into two groups (n = 6 per group): an uninjured group (control) and a 4-week CNC neuropathy group.

We performed LDF, FAG, electrodiagnostic examination (EDX), and histological examination after removing the sutures from the sciatic nerve during reoperation. This experiment measures a newly developed model of severe neuropathy in larger animals, focusing on its clinical application.

### 2.2. Clinical Study

All patients consented to participate in the clinical study, and written informed consent was obtained from all participants before surgery. This study adhered to the ethical guidelines of our institution (approval number: 3095). It included patients who underwent open CTR (OCTR) with/without simultaneous opponoplasty for severe CTS [24], as defined by EDX findings of low amplitude or absence of compound muscle action potential (CMAP) of the abductor pollicis brevis (APB) or the sensory nerve action potential (SNAP). The CTS diagnosis was based on clinical observations and electrophysiological examinations performed by specialists. Clinical observations included physical signs such as the Phalen test, Tinel sign, ring finger splitting, various maneuvers, and a history interview according to the guidelines [25]. The indication for simultaneous opponoplasty was based on the patient’s preference following informed consent. We excluded patients with recurrent or mass lesions in the carpal tunnel and those who underwent hemodialysis. FAG was performed intraoperatively after releasing the carpal tunnel and identifying the median nerve. Surgical procedures were performed by two hand surgeons (M.O. and T.Y.) at our institution.

#### 2.2.1. Fluorescein Angiography (In Vivo and Clinical Study)

In the animal study, FAG was performed following the previous report [18]. Briefly, we exposed the left sciatic nerve of the animal models and removed the silicone tubes and sutures from the rat and rabbit models, respectively.

We injected 0.4 mL of fluorescein (100 mg/mL) intraperitoneally to the rats and intravenously to the rabbits. Fluorescein in the microvascular system was visualized using a FAG filter (Scimen Design Ltd., Tokyo, Japan) with a surgical microscope (Leica M520 MS3, Leica Microsystems, Wetzlar, Germany). The recording duration was set to 10 min, with the filter placed approximately 25 cm from the object. The luminance of fluorescein distributed in the sciatic nerve was quantified using the luminance analysis software Flowinsight (Version 2.5.0) (Infocom Corporation, Tokyo, Japan). The region of interest (ROI) was set at the compression site, and peak luminance was automatically scored on a scale ranging from 0 (black) to 255 (white) points.

In this clinical study, OCTR was performed under general or local anesthesia using an air tourniquet. A skin incision was made on the palm of the carpal tunnel. The transverse carpal ligament was incised to release the median nerve. The median nerve was identified under a surgical microscope.

**Figure 4:**
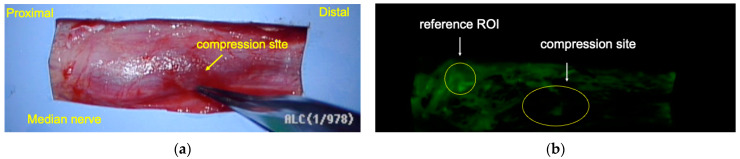
Fluorescein angiography (FAG) for a CTS patient. (**a**) The forceps are pointed at the compression site of the median nerve. (**b**) The region of interest (ROI) of the compression site and reference. CTS, carpal tunnel syndrome.

The surface temperature was maintained at 37.0 °C using warm saline solution. FAG was recorded by injecting 0.4 mL of fluorescein (100 mg/mL) intravenously 10 min after releasing the air tourniquet. The luminance of the proximal uncompressed area was set as a reference for calibration among patients to address variability in body weight and blood pressure. The ROI was defined at the compression site, and the luminance ratio (luminance of the compression site/luminance of the reference site) was calculated (Figure 4). In cases of simultaneous opponoplasty, the Camitz procedure [26] was performed following FAG.

#### 2.2.2. Electrodiagnostic Examination (In Vivo and Clinical Study)

In a rabbit model, EDX was performed following a previously reported procedure [27]. Briefly, the rabbits were anesthetized, and the sciatic nerves were identified. A NICOLET VIKING SELECT electromyogram machine (Natus Neurology Inc., Middleton, WI, USA) was used to record electrophysiological signals.

The proximal end of the compression site was stimulated using a monopolar 28-G needle electrode. The recording needle electrode was inserted into the gastrocnemius muscle, while a reference electrode was inserted into the Achilles tendon. A series of nerve stimulations with repetitively generated single pulses of 0.1 ms duration were performed until a maximal artifact-free compound muscle action potential (CMAP) motor response was elicited.

Nerve conduction studies were performed using standard procedures [25] with a Neuropack X1 electromyogram machine (NIHON KOHDEN, Tokyo, Japan). The skin temperature measured on the hand was maintained above 31 °C. The compound muscle action potential (CMAP) was recorded using a surface electrode on the abductor pollicis brevis (APB), with stimulation of the median nerve at the wrist approximately 7 cm from the palm. A series of nerve stimulations with repetitively generated single pulses of 0.1 ms duration were performed until a maximal artifact-free CMAP motor response was elicited. The sensory nerve action potential (SNAP) was recorded using a surface electrode placed approximately 2.5 cm from the wrist crease on the median nerve, and the stimulating electrode was positioned with a ring electrode on the index finger. A single pulse of 10 Hz nerve stimulation was repeatedly generated, and the average SNAP amplitude of 100 stimulations was recorded.

#### 2.2.3. Laser Doppler Flowmetry (In Vivo Study)

We used LDF (FLO-C1, Omegawave Co., Ltd., Tokyo, Japan) to monitor blood flow at the compression site. This device could measure real-time blood flow within a depth of 1 mm [28], and the probe was positioned in the middle of the compression site without applying pressure. The mean blood flow was recorded for 30 s after the waveform stabilized without artifacts.

### 2.3. Histological Examination for the Rabbit Model

We performed a histological examination of the rabbit model, as this model was not established in previous studies. The compression site of the sciatic nerve was harvested from both groups post-euthanasia. Masson’s trichrome staining was used to assess epineural fibrosis in the nerve, while toluidine blue staining was used to evaluate myelin and axons in the outer rim of the nerve [18]. Sections were observed under a microscope (BX53F; Olympus, Tokyo, Japan), and images were captured using a digital camera (DP74; Olympus, Tokyo, Japan).

### 2.4. Statistical Analysis

All data are presented as mean ± standard deviation (SD). The Kruskal–Wallis test with Bonferroni correction was used to compare LDF and FAG values between the rat model groups. The Mann–Whitney U test was used to compare the same values between rabbit models.

Relationships were determined using Spearman’s correlation coefficients, with statistical significance set at *p* < 0.05. All analyses were performed using the EZR software (version 2.7-2; Saitama Medical Center, Jichi Medical University, Shimotsuke, Japan).

## 3. Results

### 3.1. Rat CNC Model

#### Blood Flow Evaluations

**Figure 5:**
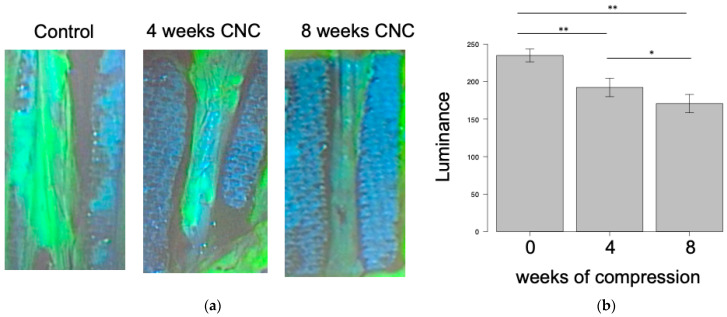
Fluorescein angiography (FAG) of rat sciatic nerve. (**a**) The luminance of FAG decreased over the weeks. (**b**) Luminance of the compression site. Significant differences were observed between the control group and the 4- or 8-week CNC neuropathy group (** *p* < 0.01) and between the 4- and 8-week CNC neuropathy group (* *p* < 0.05).

The luminance of FAG decreased over the weeks: the control group showed a luminance of 234.8 ± 8.47, the 4-week CNC neuropathy group 192.2 ± 12.3, and the 8-week CNC neuropathy group 170.7 ± 12.2. Significant differences were observed between the control group and the 4- or 8-week CNC neuropathy group (*p* < 0.01), and between the 4- and 8-week CNC neuropathy group (*p* < 0.05) (Figure 5).

The mean blood flow measured by LDF decreased over the weeks: the control group had a mean blood flow of 19.1 ± 0.93 mL/min/100 g, the 4-week CNC neuropathy group 14.7 ± 5.3 mL/min/100 g, and the 8-week CNC neuropathy group 10.9 ± 1.8 mL/min/100 g.

**Figure 6:**
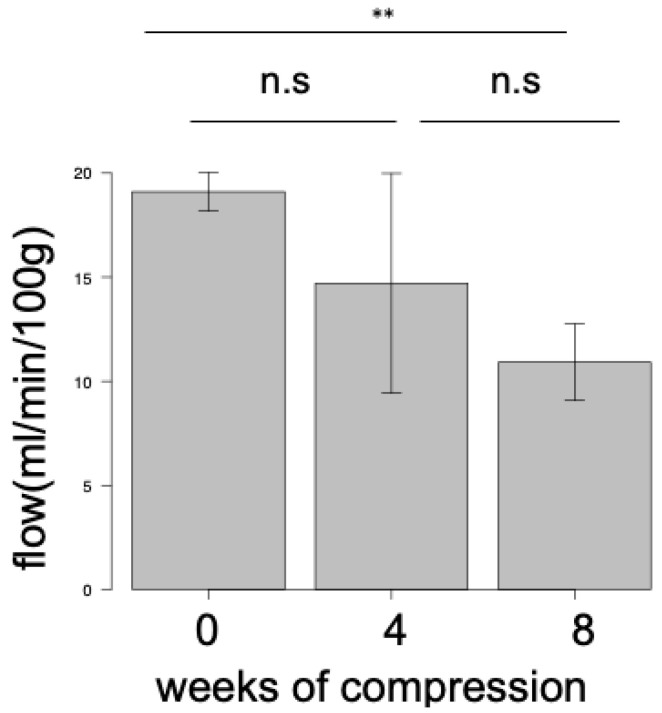
Laser Doppler flowmetry (LDF) of rat sciatic nerve. A significant difference was observed between the control group and the 8-week CNC neuropathy group (** *p* < 0.01). n.s, not significant.

Significant differences were observed between the control group and the 8-week CNC neuropathy group (*p* < 0.01) (Figure 6).

We identified a significant correlation between the luminance of FAG and the mean blood flow measured by LDF (r = 0.65; *p* < 0.01).

Based on these findings, we performed lactate dehydrogenase (LDF) and FAG assays in the rabbit model.

### 3.2. Rabbit Severe Chronic Neuropathy Model

#### Electrodiagnostic Examination

**Figure 7:**
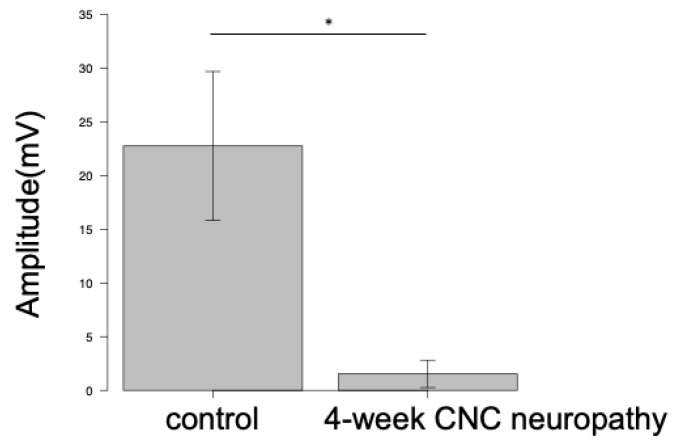
Comparison of CMAP amplitude of rabbit sciatic nerves between the control group and the 4-week CNC neuropathy group. A significant difference was observed between the control group and the 4-week CNC neuropathy group (* *p* < 0.05). CMAP, compound muscle action potential.

The amplitude of the CMAP of the gastrocnemius muscles in the 4-week CNC neuropathy group (1.53 mV ± 1.27) was significantly lower than that in the control (22.8 mV ± 6.93, *p* < 0.01) (Figure 7).

### 3.3. Histological Examination

**Figure 8:**
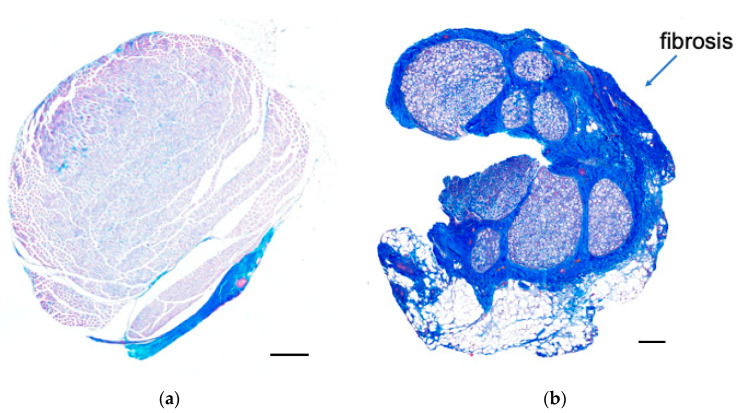
Masson’s trichrome staining of rabbit sciatic nerves indicates epineural fibrosis in the affected nerve for (**a**) the control and (**b**) after 4-week CNC neuropathy. Scale bar = 200 μm.

**Figure 9:**
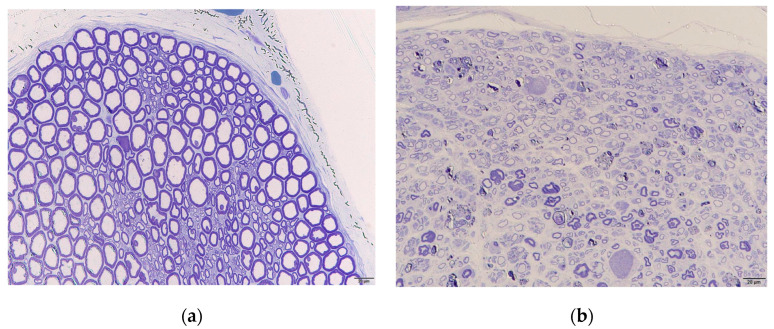
Toluidine blue staining of rabbit nerves. The morphology of the axons was deformed, and the myelin in the outer rim of the nerve was thinner in the affected nerve for (**a**) the control and (**b**) 4-week CNC neuropathy. Scale bar = 20 μm.

Masson’s trichrome staining revealed that the epineurium was thicker in the neuropathy group than in the control group (Figure 8). Toluidine blue staining also showed deformities in axon morphology and thinner myelin in the outer rim of the nerve in the neuropathy group compared to in the control group (Figure 9).

Based on these electrophysiological and histological examinations, we confirmed that this new animal model exhibited severe neuropathy with axonal damage and demyelination.

### 3.4. Blood Flow Evaluations

**Figure 10:**
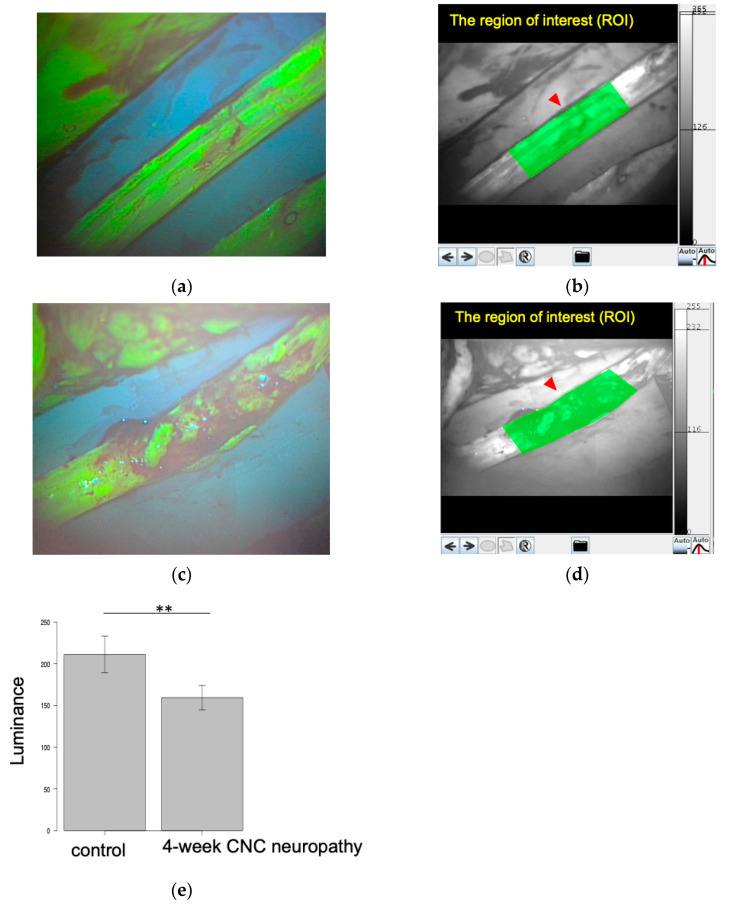
Blood flow assessment by fluorescein angiography (FAG) for rabbit sciatic nerve. Sciatic nerves of the control group (**a**,**b**) and the 4-week CNC neuropathy group (**c**,**d**) in the FAG analysis. The red arrows indicate the ROI. (**e**) Comparison of the luminance of rabbit sciatic nerves between the control and 4-week CNC neuropathy groups. A significant difference was observed between the control group and the 4-week CNC neuropathy group (** *p* < 0.01).

The luminance of the 4-week CNC neuropathy group (159.5 ± 14.6) was significantly lower than that of the control group (211.2 ± 21.9, *p* < 0.01) (Figure 10).

**Figure 11:**
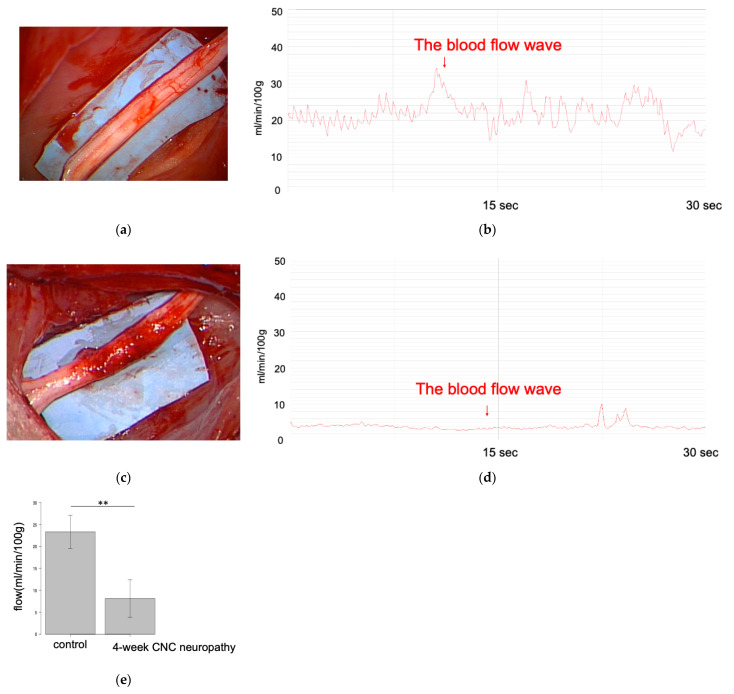
Blood flow assessment by laser Doppler flowmetry (LDF) for rabbit sciatic nerve. Sciatic nerves in the control (**a**) and 4-week CNC neuropathy groups (**c**). Blood flow was recorded and calculated using FLO-C1 in the control (**b**) and 4-week CNC neuropathy groups (**d**). (**e**) Comparison of the blood flow of rabbit sciatic nerves between the control group and the 4-week CNC neuropathy group. A significant difference was observed between the control group and the 4-week CNC neuropathy group (** *p* < 0.01).

Similarly, the mean blood flow measured by LDF in the 4-week CNC neuropathy group (23.3 mL/min/100 g, ±3.8) was lower than that of the control group (8.11 mL/min/100 g, ±4.3, *p* < 0.01) (Figure 11). There was no significant correlation between the luminance of the FAG and the mean blood flow measured by LDF (r = 0.83; *p* = 0.06).

We identified significant correlations between CMAP amplitude and FAG luminance (r = 0.69; *p* < 0.05). However, no significant correlation was observed between the amplitude of the CMAP and the mean blood flow measured by LDF (r = 0.6; *p* = 0.24).

### 3.5. Clinical Study

In the study, 31 patients were included, with a mean age of 70.9 (±11.3 years), and 19 patients (61.3%) were women. Fourteen patients (45.2%) underwent simultaneous opponoplasty (Table 1). The mean preoperative SNAP amplitude was 1.21 μV (±2.7). The mean luminance ratio was 0.64 (±0.31). No significant correlation was observed between the luminance ratio and SNAP amplitude (r = 0.34; *p* = 0.06).

The mean preoperative amplitude of APB-CMAP was 3.02 mV (±4.3). The mean luminance ratio was 0.64 (±0.31). We identified a significant correlation between the luminance ratio and CMAP amplitude (r = 0.48; *p* < 0.01).

## 4. Discussion

We demonstrated that fluorescein angiography (FAG) and laser Doppler flowmetry (LDF) could effectively reflect blood flow reduction over time in a rat model of CNC. Subsequently, we applied these methods to a newly developed severe chronic neuropathy rabbit model, where both FAG and LDF showed significant reductions in blood flow compared to controls. Additionally, we observed a correlation between electrodiagnostic findings and FAG but not with LDF. In the clinical setting, FAG correlated with preoperative CMAP amplitude in patients with severe CTS, consistent with the results of the rabbit model. Our findings demonstrate that FAG is a reliable diagnostic method for visualizing reduced blood flow, particularly in severe neuropathy models, including both large animal and human clinical cases. The observed correlations between FAG findings and electrodiagnostic findings in both the sciatic nerve neuropathy model and clinical studies involving patients with severe CTS reinforce the utility of FAG. This consistency across different settings suggests that FAG can be effectively applied to various types of neuropathies, providing a valuable tool for both experimental research and clinical practice.

Microvessels in the epineurium extend longitudinally and branch transversely through the perineurium, creating a network of primary capillaries [29]. Peripheral nerve injury due to chronic CNC leads to a sequence of pathological events that result in decreased blood flow [30,31,32]. Previous studies have demonstrated that the mechanism begins with mechanical nerve compression, leading to edema and subsequent fibrosis of the epineurium and perineurium [5,6]. This fibrosis causes further mechanical stimulation of adjacent tissues and disruption of blood flow within the epineurium, thereby increasing the internal pressure within the nerve [5,6]. These changes cause marked thinning of the myelin sheath and Schwann cell pathology, impairing electrical signal conduction [5,33].

Hama et al. found that FAG effectively measures the decrease in neural blood flow associated with these pathological stages [18]. Fluorescein was observed to be predominantly distributed within the epineurium, vessels in the epineurium, and endoneurial microvessels, which were significantly reduced in the CNC neuropathy group compared with the control group. The study also discussed the potential of FAG as an early marker for detecting neuropathy, as it revealed deterioration in luminance before observable demyelination and axonal degeneration. This suggests that FAG can reduce blood flow at earlier stages of the neuropathic process. Applying this method to a rat model showed a significant reduction in luminance compared with the control group, starting from the 4-week CNC neuropathy group. Additionally, there was a significant decrease in luminance between the 4- and 8-week CNC neuropathy groups, suggesting that FAG can both be used as an early marker and assessing severity progression.

LDF is a noninvasive method used to continuously measure microcirculatory blood flow by analyzing the Doppler shift of low-power laser light scattered by moving blood cells [34]. Previous studies have demonstrated its usage to measure blood flow changes in various clinical and experimental settings, including the central nervous system [35,36], peripheral nerves [17], dental pulp [37], and skin [38]. Furthermore, LDF was used to evaluate blood flow to the median nerve during carpal tunnel release surgery [17], demonstrating its effectiveness in monitoring real-time changes in nerve blood flow during surgery. In another study, LDF was used to measure the spinal cord blood flow in patients with syringomyelia before and after shunt surgery, showing that spinal ischemia plays a crucial role in the pathophysiology of syringomyelia [36]. These findings further support the effectiveness of LDF in detecting changes in neural blood flow. In our study, we extended these findings by applying LDF to CNC rat and rabbit models. We found that blood flow measured using LDF significantly decreased with the progression of neuropathy. This result aligns with the outcomes observed using FAG.

Notably, the correlation between FAG and LDF tends to decrease when transitioning from rats (r = 0.65; *p* < 0.01) to rabbits (r = 0.83; *p* = 0.06). This discrepancy is likely due to the greater depth of the rabbit sciatic nerve, which exceeds the 1 mm measurement depth of the LDF [28]. In the rabbit model, the LDF waveform appeared flattened compared with that in the rat model, possibly reflecting the limited ability of LDF to penetrate deep tissue. Despite these limitations, LDF demonstrated significant changes in blood flow in the rabbit model, although its correlation with neuropathy severity was less clear than that of FAG. Conversely, FAG showed a strong correlation with the severity of neuropathy, even within larger anatomical structures. This suggests that FAG has superior detection capability for evaluating perfusion in deeper tissues of severe CNC neuropathy models. The endothelial cells of endoneurial microvessels are interconnected by tight junctions, forming a blood–nerve barrier (BNB) [39]. The BNB is crucial in controlling the internal environment of peripheral nerves [18,40], and it is located in the perineurial and endoneurial microvessels [41]. Conversely, epineurial microvessels lack the BNB and are highly susceptible to compression trauma, ischemia, and anatomical injury compared to endoneurial vessels [39]. This characteristic of neural microvasculature may emphasize the importance of detecting residual neural blood flow from deeper layers in severe CNC neuropathy models. This enhanced detection capability of FAG suggests that it may be a more reliable tool than LDF in assessing the severity of neuropathy, particularly in cases where deeper tissue evaluation is necessary.

In the clinical study, we observed a significant correlation between FAG luminance ratio and preoperative CMAP amplitude in patients with severe CTS. This finding aligns with the results from our rabbit model, where FAG showed a strong correlation with the EDX findings. Our clinical results suggest that FAG effectively evaluates nerve blood flow post-decompression during CTS surgery. The correlation between FAG luminance and CMAP amplitude indicated that FAG reflects the severity of nerve damage. This capability is advantageous for intraoperative assessment, enabling surgeons to confirm the adequacy of real-time decompression.

This study has several limitations.

First, we used the sciatic nerve model in animal experiments, while the clinical research focused on CTS, which affects the median nerve. The sciatic nerve model provides valuable insights into CNC neuropathies, with a previous study demonstrating significant changes in myelination and Schwann cell proliferation, both critical responses to CNC [22]. Therefore, the sciatic nerve model is widely used as a CNC model. Consequently, we chose the sciatic nerve model as the initial step in our animal experimentation. Second, the sample size was relatively small, limiting the generalizability of the results. Future studies should increase the sample size to collect robust data and enhance the reliability of their findings. Third, this study did not include a sham surgery group, which could have influenced the results because of postoperative adhesions. Its introduction at different time points could have enhanced the accurate assessment of the adhesion effects. However, this study focused on clinical applications for patients with carpal tunnel syndrome, excluding reoperation cases; hence, sham surgery was not performed. Fourth, blood flow was assessed post-decompression during surgery, limiting the clinical relevance to confirming the adequacy of the decompression area and assessing severity in real-time. In clinical practice, future studies should include large-scale investigations to determine whether intraoperative blood flow assessments can predict long-term outcomes. The final limitation concerns the subjectivity of measurement methods. To address these limitations, we implemented a standardized and simplified surgical approach, and the measurements were automated using software. LDF measurements can be influenced by factors such as probe movement, positioning, and bleeding [17]. However, LDF signals are reproducible and can be readily acquired in clinics and laboratories [17]. For FAG in clinical applications, a calibration method using references was adopted, considering the greater variability between patients in factors such as intraoperative blood pressure, hemoglobin levels, and body weight compared to animal models. In the future, standardization of these measurement methods is necessary to address procedural variability and enhance reproducibility.

## 5. Conclusions

In this study, FAG demonstrated significant correlations with electrodiagnostic findings in both animal models and human clinical cases, particularly in severe neuropathy scenarios. In animal studies, FAG effectively visualized blood flow reductions in both rat and rabbit models of chronic nerve compression neuropathy, showing a strong correlation with neuropathy severity, even in larger anatomical structures. In clinical studies, FAG was significantly correlated with preoperative CMAP amplitudes in patients with severe CTS, further reinforcing its potential as a reliable intraoperative diagnostic tool. These findings suggest that FAG may offer reliable detection capabilities in evaluating blood flow, particularly in deeper tissues and severe neuropathy models. Future research should focus on further validating FAG’s reliability in larger clinical populations and exploring its potential for predicting long-term outcomes in neuropathy patients.

## Figures and Tables

**Table 1:** Demographic data of the study participants.

Characteristic (Total, n = 31)	Value
Age	
Years (mean ± SD)	70.9 ± 11.3
Sex	
Female	19 (61.3%)
Male	12 (38.7%)
Electrodiagnostic Examination	
Amplitude (mV) of APB CMAP, mean ± SD	3.02 ± 4.3
Amplitude (μV) of SNAP, mean ± SD	1.21 ± 2.7

CMAP, compound muscle action potential; SNAP, sensory nerve action potential; APB, abductor pollicis brevis.

## Data Availability

The data that support the findings of this study are available from the corresponding author upon reasonable request.
